# Evaluation of privacy protection methods of public service advertising visual design in the perspective of artificial intelligence internet of things

**DOI:** 10.1371/journal.pone.0308732

**Published:** 2024-12-02

**Authors:** Xujun Tang

**Affiliations:** College of Art and Design, Guilin Tourism College, Guilin, Guangxi, China; The University of Dodoma, UNITED REPUBLIC OF TANZANIA

## Abstract

With the widespread application of monitoring technology and network storage technology, there are some privacy issues in the process of public service advertising visual design and the process of pushing public service advertising visual design works. Researching the privacy protection method of public service advertising visual design is of great significance to protecting the privacy data security of public service advertising visual designers and the personal privacy of other citizens. This paper proposes a digital watermark privacy protection method and a public service advertising visual design work push privacy protection method, and conducts a privacy protection experiment study on public service advertising visual design. The digital watermark privacy protection method and the public service advertising work push privacy protection method are used to provide an innovative solution for public service advertising privacy protection. The study shows that the evaluation score of the digital watermark privacy protection method is 11.05% higher than that of the traditional passive privacy protection method; the average feasibility evaluation score and the average effectiveness evaluation score of the privacy protection method for the public service advertising visual design work push by experts are 86.8 and 90.66 respectively. Combining the perspectives of artificial intelligence and the Internet of Things, this paper proposes a digital watermark privacy protection method, emphasizing practicality, and providing new ideas for privacy protection of public service advertising visual design.

## 1.Introduction

The extensive and deep application of network storage technology makes people face some privacy security problems. For example, in the online environment, privacy problems may arise in the process of public service advertising visual design, and the privacy data of public service advertising visual designers may also be exposed. In the process of personalized recommendation of public service advertising visual design works, there are also privacy security issues. The privacy problems in the visual design of public service advertisements are concerned about the risk of privacy data leakage under the Internet of Things platform, and the privacy protection method of digital watermarking is proposed. In the design of public service advertisement, privacy protection strategies mainly include digital watermarking technology and expert evaluation push method. Digital watermarking is applied to AIoT cloud platform to ensure the security of private data. Expert evaluation ensures the privacy of public service advertising works by evaluating the feasibility and effectiveness. Therefore, based on the AIoT perspective, this paper analyzed the privacy protection methods on the visual design of public service ads, so as to protect the privacy data security of stakeholders and hope to provide valuable reference for relevant research. In this paper, the privacy protection methods of digital watermarking and public service advertising works are put forward to deal with the privacy problems in public service advertising visual design and provide effective solutions for maintaining the privacy safety of designers and audiences. This paper studies privacy protection methods based on the perspective of artificial intelligence and the Internet of Things, provides an innovative solution for privacy protection in the visual design of public service advertisements, fills the gap in privacy protection research in the advertising field, and has important theoretical and practical value.

Many scholars have analyzed privacy protection methods. Yin Chunyong proposed a location privacy protection method meeting the differential privacy constraint to protect the location data privacy and maximize the utility of data and algorithms in industrial IoT [1]. Thomas Winkler believed that ubiquitous networked sensors posed a huge challenge to privacy protection and introduced an objective evaluation framework to evaluate this protection method [2]. Liu Tianen believed that the privacy protection of IoT was very important in the participatory awareness framework. He studied how to protect the privacy information of participants in insecure network channels by using cryptography based methods to achieve dynamic and real-time awareness tasks [3]. Gu Ke provided a framework for privacy protection of location data mining, and proposed a privacy protection scheme for location data records based on differential privacy mechanism [4]. In order to prevent the disclosure of sensitive information in cloud storage, Liu Jie proposed a density based K anonymous privacy protection clustering method [5]. Boerman Sophie C believed that personal management and protection of online privacy became an important part of daily life, and investigated online privacy protection behavior using the theory of protection motivation [6]. Martin Kelly D. studied the theoretical views and empirical findings on data and information privacy, and introduced the future research topics embodying multidimensional methods. This method integrated many interrelated issues in contemporary marketing privacy issues [7]. Combined with the complexity and particularity of railway business, Zhang Weizhen put forward the technical system of railway data security and privacy protection, analyzed the demand and risk of railway data security and privacy protection, and adopted the method of classifying railway data and protecting the whole life cycle data security [8]. The above scholars have studied the privacy protection methods and put forward some valuable suggestions, but they rarely study the privacy protection methods of advertising visual design. The above scholars put forward diversified methods in privacy protection, covering the fields of location privacy, perception task, differential privacy and so on. However, under the background of advertising visual design, the research is relatively limited. From the perspective of artificial intelligence Internet of Things, this paper innovatively discusses digital watermarking and push privacy protection methods, which fills the research gap of privacy protection in advertising field.

Many scholars have studied advertising and privacy. Kox Henk analyzed the invasion of personal privacy caused by advertising, and studied the benefits of privacy protection strategies to consumers and websites [9]. Segijn Claire M. studied the personal privacy problems caused by synchronous advertising to consumers. The conclusion was that for people who paid more attention to privacy issues, the effect of synchronous advertising might be poor [10]. Liu Dongxiao proposed an intelligent advertising network with privacy protection responsibility based on blockchain [11]. Lina Lia Febria studied the impact of personalized advertising on consumers’ purchase intention, and discussed the privacy problems caused by personalized advertising [12]. Jiang Jinghua proposed a secure billing scheme to enable the advertising network to charge advertisers in a way that protected privacy, so as to protect personal privacy and security [13]. To sum up, many scholars have participated in the research on advertising and privacy.

With the rapid development of modern information technology, the visual design of public service advertisements is gradually facing severe challenges in privacy protection. In existing research, many scholars have proposed privacy protection methods based on the combination of artificial intelligence and the Internet of Things, and explored cutting-edge technologies and applications in this field from different perspectives. Based on the integration of smartphone network architecture and data security technology, some studies have proposed solutions to mitigate malicious behavior in non-profit organizations [14]. The study emphasizes that by combining smartphone network architecture with data security technology, the level of data protection can be effectively improved and privacy leakage can be prevented. This method has certain reference significance for privacy protection in the visual design of public service advertisements. Combining the development of AI, IoT and 5G technologies, some studies have conducted a trend and prospect survey on the next generation of smart grids [15]. This study demonstrates the application prospects of AI and IoT technologies in smart grids, and provides a new perspective for the intelligent push and privacy protection of public service advertisements. Through the deep integration of AI and IoT, real-time monitoring and intelligent analysis of public service advertisement data can be achieved, effectively improving the efficiency and reliability of privacy protection. Artificial neural network technology also plays an important role in privacy protection. For example, some studies use ANN to analyze steganographic images, improving data anonymity and privacy protection [16]. In addition, in credit card fraud detection, neural network integration and feature engineering methods have been shown to significantly improve detection accuracy [17]. The application of these technologies in public service advertisements can achieve efficient protection of sensitive information through the training and optimization of deep learning models. Further research shows that the accuracy of credit card default prediction can be improved by using the stacked sparse autoencoder method [18]. This technology can also be applied to the privacy protection of public service advertisements, and through deep mining and learning of data features, accurate protection and management of user privacy can be achieved. In edge-assisted SDWSN, AI-based intrusion detection and prevention technology also performs well [19]. By modifying the cellular structure, the study proposed an improved intrusion detection and prevention scheme, which provides a new technical path for the privacy protection of public service advertisements. This scheme not only improves the security of the system, but also enhances the detection and response capabilities of potential threats. Combined with the above research results, it can be seen that the application of AI and IoT technologies in the privacy protection of public service advertisements has broad prospects. By drawing on advanced technologies and methods from different fields, a more comprehensive and efficient privacy protection scheme can be provided for the visual design of public service advertisements, further promoting technological progress and application development in this field.

This paper discussed the application of digital watermarking in the privacy protection of public service advertising visual design, and analyzed the privacy protection methods in the push of public service advertising visual design works. From the experts’ evaluation of digital watermark privacy protection methods and traditional passive privacy protection methods, and the evaluation of privacy protection methods for public service advertising visual design works, the experimental research on privacy protection of public service advertising visual design was carried out. The privacy protection method of digital watermarking includes applying digital watermarking technology in the private data storage system of AIoT cloud platform. The privacy protection method of public service advertising works involves expert evaluation, and the practicability of this method is verified by evaluating its feasibility and effectiveness. Experiments show that the digital watermark privacy protection method scores higher than the traditional method, and the push privacy protection method is recognized by experts in feasibility and effectiveness.

## 2.Element evaluation of public service advertising visual design

**Fig 1:**
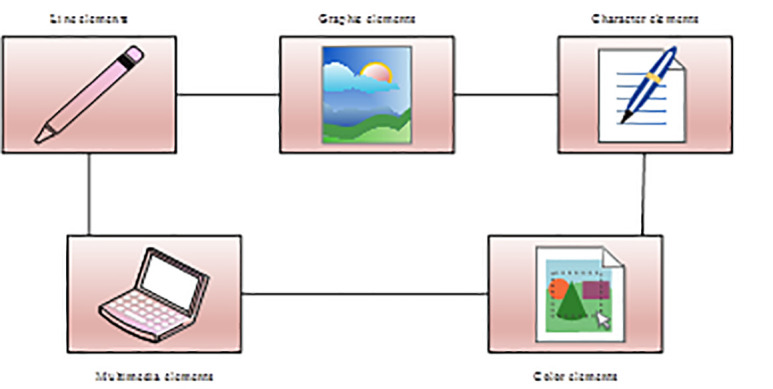


The elements of public service advertising visual design mainly include line elements, graphic elements, character elements, color elements and multimedia elements, as shown in Fig 1 Analysis of elements of visual design of public service advertisements.

### (1) Line element

Line is the result of the movement of points in two-dimensional space, which is the opposite of the basic painting element point. The line has the function of dividing the picture according to the artistic aesthetic principle in the public service advertisement picture. Its image in the public service advertisement visual design includes two kinds: One is a conceptual and abstract line, and the other is a concrete line. The conceptual and abstract lines express virtual and abstract concepts, and the figurative lines usually represent substances with solid bodies. Compared with commercial advertisements, the lines in public service advertisements play less decorative functions, and the lines in public service advertisements often pay more attention to the visual segmentation function and emotional expression function played by lines. For example, the nest built by the lines of the public service advertisement “*Caring for the Empty Nester*” fully reflects the loneliness of the empty nester.

### (2) Graphic element

The functions of graphic elements in public service advertising design are mainly reflected in the following aspects: Graphic elements can make the audience clear about the visual communication theme of public service advertising, so that the audience can easily understand the content of public service advertising; the visual effect of graphic elements is conducive to attracting the attention of public service advertising audience, which also helps the audience to read the character elements of public service advertising. Graphic elements are indispensable in the visual design elements of public service advertisements. It is easy to be captured by human vision, with high recognition and strong symbolic significance.

### (3) Character element

There are two types of character elements: text and symbol. Text and symbol are important ways to convey advertising information to the public service advertising audience, which are usually used to assist the visual expression of graphic elements. In addition, there are also some public service advertisements that use character elements as the visual communication subject. For example, the visual design of “*Filial Piety*”, a public service advertisement, takes “filial piety” and “old” as the main visual communication subjects, which conveys the theme of filial piety and caring for the elderly.

### (4) Color element

Color element is a powerful tool to attract public service advertising audience. From the perspective of the visual laws of advertising colors, public service advertising audiences usually think that the colors with high attention and visual impact are solid colors, warm colors and bright colors. It is generally believed that the colors with low attention and weak visual impact include cold colors, low chroma colors and dark colors. The eye-catching degree of color elements is also affected by color collocation. For example, if the background color is similar to the graphic color, the degree of attention of color elements is low. If the background color and graphic color are different, the degree of attention of color elements is high. Color elements not only affect the visual sense of public service advertising audience, but also affect the emotional changes of public service advertising audience. Of course, due to the social and cultural differences between different regions, different regions have different interpretations of color.

### (5) Multimedia element

Different from line elements, graphic elements, character elements and color elements, multimedia elements are not the basic elements in public service advertising design. Multimedia elements, as elements of public service advertising visual design, are the needs of the real social environment faced by contemporary public service advertising design, because they are important factors affecting the visual communication of public service advertising.

## 3.Principles of public service advertising visual design

**Fig 2:**
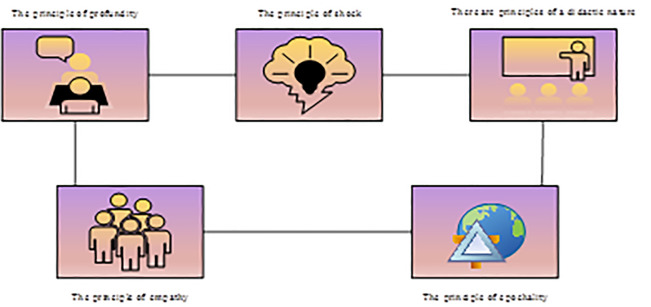


The principles of public service advertising visual design include the principles of profundity, shock, preaching, timeliness and resonance, as shown in Fig 2 Principles of visual design of public service advertisements.

### (1) Principle of profundity

Although public service advertising does not add commercial value, the social benefits brought by public service advertising are far beyond the scope of commercial advertising. The purpose of public service advertising visual design is not to export the concept of commercial consumption to the public but to expose the social problems. As a mirror reflecting the human society, public service advertising does not simply stop at the surface, and it should reveal the root causes of social problems and the essential characteristics of human social problems. Public service advertising adheres to the principle of profound visual design, which can lead people to think deeply about social problems and help to achieve twice the result with half the effort.

### (2) Shock principle

Public service advertising is different from commercial advertising. The visual design of public service advertising should follow the principle of solemnity and seriousness. The visual design language it uses can not be lightly modified, and it should make the public service advertisement audience receive a strong visual impact through the use of shocking visual design language. Therefore, the public service advertising visual design should follow the principle of shock, and create a shock visual design image, so as to attract the attention of public service advertising audience and more effectively reflect the values contained in public service advertising.

### (3) Timeliness principle

Public service advertising is like a teacher who follows good guidance and conveys a didactic language to the public service advertising audience. The visual designers of public service advertisements should follow the principle of preaching and continue to develop public welfare undertakings, which should assume the responsibility of conveying correct values to the public.

### (4) Epochal principle

As human society faces different problems in the process of sustainable development, the theme of public service advertising visual design is not static. The visual design of public service advertisement should keep pace with the times and pay attention to the main problems exposed in human society and the emerging social problems.

### (5) Resonance principle

If the public service advertisement visual designer wants to achieve the purpose of conveying the correct value concept to the public, the principle of resonance should be adhered to, and the creation source should be extracted from the life of the public. In the content of public service advertising visual design works, resonance thinking is added to make the public have resonance feelings.

## 4.Application of digital watermarking in the privacy protection of public service advertising visual design from the perspective of AIoT

### (1) Private data AIoT cloud platform storage system

Digital watermarking is a kind of hidden information embedded in images or multimedia content, which is used for identity verification or copyright protection. In public service advertisements, digital watermarking can be used to identify and protect visual design and ensure the authenticity of works. However, digital watermarking is not absolutely secure, which may be affected by image processing and cropping, and some attacks may crack the watermark. We should balance the privacy protection effect of digital watermarking with the possible visual impact introduced in advertising design to ensure a balance and best practices.

**Fig 3:**
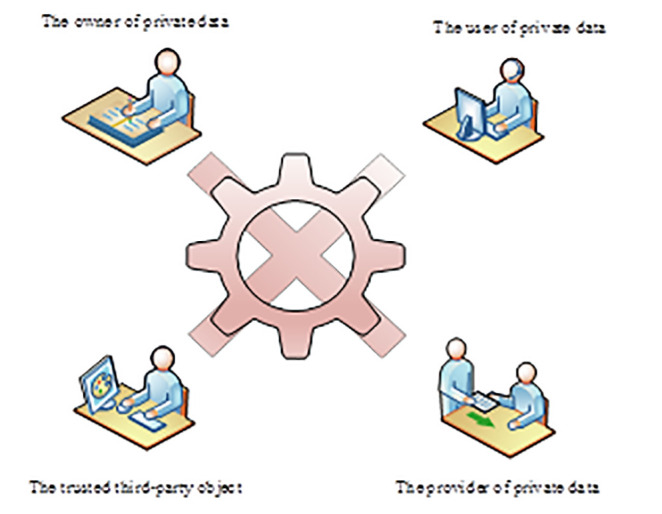


Although IoT provides a good interactive environment for people, it is more vulnerable to network attacks. Network attackers can attack multiple targets only by finding one IoT vulnerability [20,21]. Faced with this situation, Artificial Intelligence (AI) can play a certain role. The application of AI technologies such as machine learning and neural network in IoT can effectively identify and monitor each node in IoT, which improve the security of IoT [22]. Therefore, this paper studies the private data storage system based on AIoT cloud platform. The AIoT cloud platform storage system for private data using digital watermarking technology is shown in Fig 3 Privacy data AIoT cloud platform storage system.

There are four parts in the privacy data storage system, namely, the owner of privacy data, the user of privacy data, the provider of privacy data, and the trusted third-party object. The owner of private data can use encryption algorithm to encrypt the private data, and store the processed encrypted data into the database of the private data provider. Users of private data must meet the agreed access conditions when they need to access private data. They must have the authorization of the privacy data owner to access the encrypted data, and then restore the encrypted data to the original data. The privacy data provider is responsible for providing storage and retrieval services to users and verifying the rights of privacy data users to provide corresponding services according to their rights. The trusted third-party object is the key generation center. The encryption and decryption of private data are managed by generating public parameters, master keys and private keys. In the privacy protection of public service advertising visual design, before uploading the public service advertising visual design digital works, the provider of the public service advertising visual designer’s privacy data would verify whether the advertising works comply with the upload regulations by a trusted third party. If it meets the requirements, it would be uploaded to the database of the privacy data provider. When other online users need it, the trusted third party object would provide the watermark encryption service to protect the data privacy of the public service advertising visual designer. If the public service advertising visual designer is a user of private data, a trusted third party would embed the watermark information into the private data and then forward it to the public service advertising visual designer to protect the privacy of other network users’ personal data.

### (2) Application of attribute encryption algorithm in privacy data protection

The attribute encryption algorithm is applied to the AIoT storage system of private data, which helps to support the AIoT storage system to encrypt multiple private data at the same time and improve the efficiency of privacy data protection. It is assumed that j is the order of two cyclic groups and j is prime. The formula is as follows:

$$\Delta_{p,T}(\chi )=\underset{q\in T,p\neq q}{\Pi }\frac{\chi -p}{p-q}(\mathrm{mod}\;j)$$ (1)

Among them, $\Delta_{p,T}(\chi )$ is the Lagrangian parameter.

It is assumed that the size of attribute set is c, and the maximum size of encrypted private data is m. The functions are defined as follows:

$$S(\chi )=l^{\chi^{m}}\overset{m+c+1}{\underset{p=1}{\Pi }}s_{p}^{\Delta_{p,M(\chi )}}$$ (2)

Among them, *l* is a random element in a cyclic group.

The public parameters and master key are composed as follows:

$$PK=\{h_{1},h_{2},...,h_{m},l,s_{1},s_{2},...,s_{m+c+1}\}$$ (3)

$$MK=\{v_{1},v_{2},...,v_{m-1}\}$$ (4)

The keys generation of the attribute sets are as follows:

$$if\;p=0,1,...,|\omega^{\prime }|-1\;E_{p}=l^{j(p)}S{\left(p\right)}^{u_{p}}\;e_{p}=h^{u_{p}}$$ (5)

$$if\;q=1,2,...,n\;E_{q}^{\prime }=l^{j(\varepsilon_{q})}S{\left(q\right)}^{u_{q}}\;e_{p}^{\prime }=h^{u_{q}}$$ (6)

The format of ciphertext is as follows:

$$CT=\{{\left(F_{P}=S{\left(p\right)}^{t}\right)}_{p\in v},{\left(F_{q}^{\prime }=S^{\prime }{\left(q\right)}_{q}^{t}\right)}_{q\in \Lambda },{\left(K_{w}=N_{w}V_{w}^{t}\right)}_{w=0}^{m^{\prime }-1},F^{\prime \prime }=h^{t},\omega \}$$ (7)

The decryption formula of ciphertext is as follows:

$$N_{w}=K_{w}\underset{p\in T}{\Pi }{\left(\frac{f(e_{p},F_{P})}{f(E_{p},F_{P}^{\prime \prime })}\right)}^{\Delta_{p,T(w)}}\underset{p\in \Lambda^{\prime }}{\Pi }{\left(\frac{f(e_{q}^{\prime },F_{q}^{\prime \prime })}{f(E_{q}^{\prime },F_{q}^{\prime \prime })}\right)}^{\Delta_{p,T(w)}}$$ (8)

In the method of digital watermark privacy protection, more complex statistical analysis is adopted, including deep mining and analysis of privacy data, in order to improve the effect and reliability of privacy protection.

## 5.Privacy protection in the push of public service advertising visual design works

Although the push of public service advertising visual design works has a strong economic purpose different from that of commercial advertising, the personalized push of public service advertising visual design works would also bring users a lot of trouble. For the purpose of spreading the positive energy of the society and the method of improving the communication efficiency, the public service advertisement releasers would choose to improve the delivery accuracy of public service advertisements and select personalized advertising recommendation methods. However, in order to achieve personalized advertising recommendation, public service advertising would inevitably infringe on personal privacy. The application of monitoring equipment makes personal physical activity information exposed to the public, and the in-depth application of network storage technology also leads to the disclosure of personal privacy data. The public service advertiser can use AI, IoT and other technologies to analyze various personal information, so as to develop an accurate public service advertising plan. Faced with this situation, this paper studies the privacy protection methods for the push of public service advertising visual design works.

### (1) Human perspective

From the human perspective, there are three ways to achieve personal privacy protection: cultivation of the sense of responsibility, enhancement of the privacy data protection literacy and strengthening of the supervision. The cultivation of responsibility awareness means that network operators should respect personal privacy data and assume the responsibility of protecting personal privacy data. The enhancement of privacy data protection literacy refers to improving the individual’s ability to protect their own privacy, so that individuals can use legal weapons to protect their own privacy. The strengthening of supervision refers to the establishment of a privacy data protection regulatory agency to regulate individual privacy violations under the network environment.

### (2) Technical perspective

There are privacy data risks in the IoT environment. The use of relevant technologies can reduce the risk of privacy data leakage and protect personal privacy [23,24]. This paper proposes a privacy protection method to respond to the push of public service advertising visual design works from a technical perspective: Firstly, the data management of IoT devices is strengthened and the open standards for personal data are set to enable individuals to freely choose whether to open data [25,26]. Secondly, the data feedback ability of IoT equipment is strengthened, so that individuals can timely and accurately understand the collection of their own data by IoT equipment [27,28]. Thirdly, anonymous and encrypted methods are used to ensure the security of personal privacy database [29]. Fourthly, in the production stage of AI products, the protection of personal privacy data is set as a basic rule. The automatic upload of personal data is prohibited, and AI products are checked regularly to reduce the risk of privacy data disclosure.

## 6.Experimental investigation of privacy protection of public service advertising visual design

This paper first conducted three interviews in N City, and investigated the privacy protection awareness of public service advertising visual designers and public service advertising audiences through interviews. Digital watermarking privacy protection method and public service advertisement push privacy protection method are evaluated. 50 public service advertising visual designers and 50 public service advertising audiences are randomly selected for each of the three interviews. Five experts are invited to compare and evaluate the digital watermark privacy protection method and the traditional passive privacy protection method. Finally, the five experts evaluate the privacy protection method in the push of public service advertising visual design works. The basic characteristics of the respondents are shown in Table 1.

**Table 1:** Basic characteristics of respondents.

	Visual designer of public service announcements	Public Service Announcement audience
Mean age (years)	42.56	43.69
Male respondents (person)	79	83
Female respondents (person)	71	67

As shown in Table 1, the average age of the public service advertising visual designers participating in the interview is 42.56 years old, and the average age of the public service advertising audience participating in the interview is 43.69 years old. Among the public service advertisement visual designers who participated in the interview, there are 79 males and 71 females; among the public service advertisement audiences who participated in the interview, there are 83 males and 67 females.

### (1) Public service advertisement visual designer and public service advertisement audience’s privacy protection consciousness

**Fig 4:**
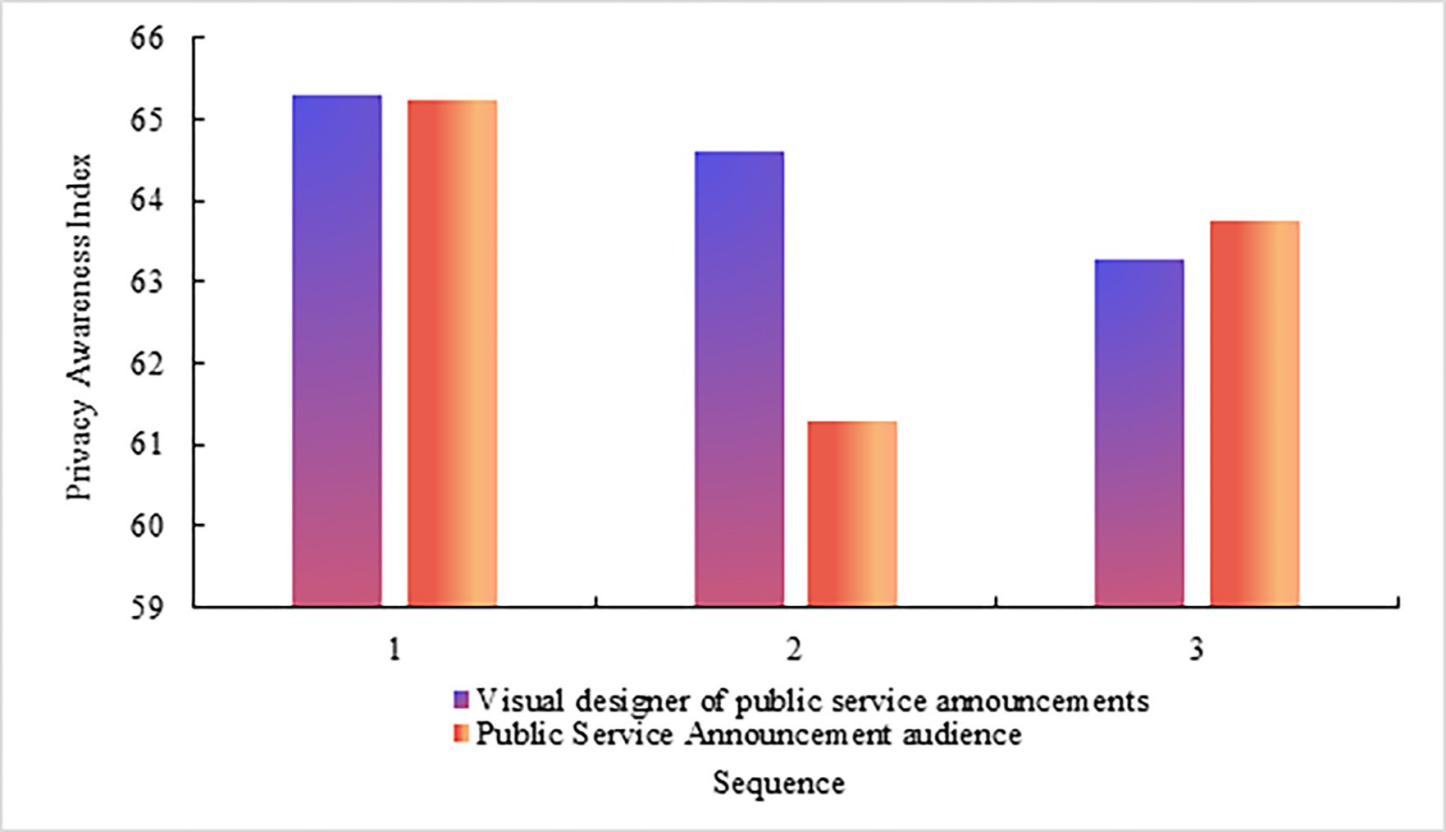


Through the interview, the privacy awareness index of the public service advertisement visual designer and public service advertisement audience is obtained. The value range of the privacy awareness index is 1–100, as shown in Fig 4 The privacy awareness of public service advertisement visual designers and public service advertising audiences.

As shown in Fig 4, in the first interview, the privacy awareness index of the public service advertisement visual designer is 65.29, and the privacy awareness index of the public service advertisement audience is 65.23. The gap between the two is small. In the second interview, the privacy awareness index of the public service advertisement visual designer is 64.59, and the privacy awareness index of the public service advertisement audience is 61.29. In the third interview, the privacy awareness index of the public service advertisement visual designer is 63.27, and the privacy awareness index of the public service advertisement audience is 63.74.

### (2) Respondents’ evaluation of digital watermark privacy protection methods and public service advertisement push privacy protection methods

**Fig 5:**
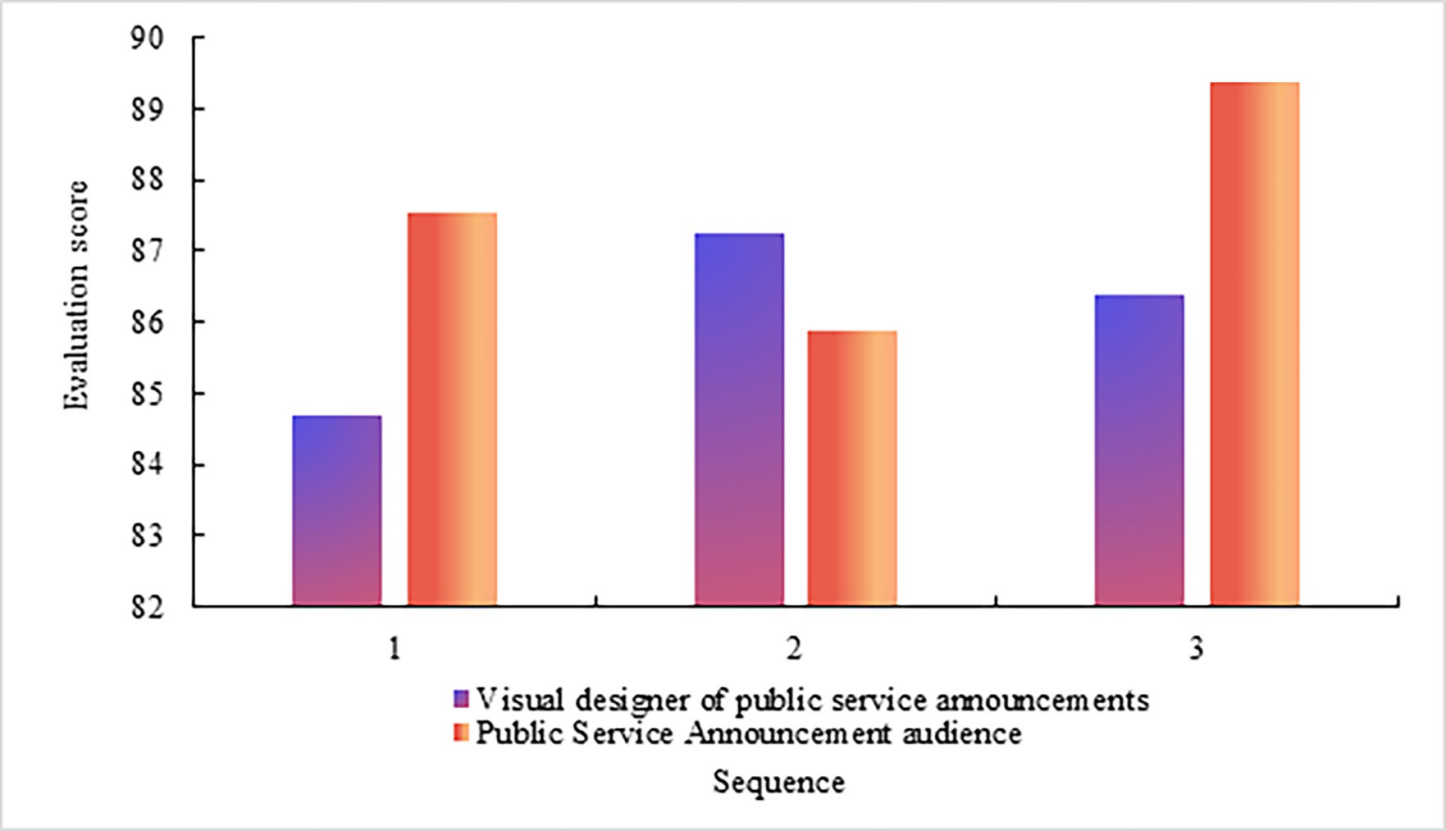


The digital watermark privacy protection method is introduced to the public service advertisement visual designer, and the public service advertisement visual designer’s evaluation of the digital watermark privacy protection method is recorded. At the same time, the public service advertising push privacy protection method is introduced to the public service advertising audience, and the public service advertising audience’s evaluation of the public service advertising push privacy protection method is recorded. The range of evaluation scores is 1–100, as shown in Fig 5 Respondents’ evaluation of the privacy protection method of digital watermark and the privacy protection method of public service advertisement pushing.

As shown in Fig 5, in the first interview, the public service advertisement visual designer’s evaluation score of the digital watermark privacy protection method is 84.69, and the public service advertisement audience’s evaluation score on the privacy protection method of public service advertisement push is 87.52. In the second interview, the public service advertisement visual designer’s evaluation score of the digital watermark privacy protection method is 87.26, and the public service advertisement audience’s evaluation score on the privacy protection method of public service advertisement push is 85.88. In the third interview, the public service advertisement visual designer’s evaluation score of the digital watermark privacy protection method is 86.39, and the public service advertisement audience’s evaluation score of the public service advertisement push privacy protection method is 89.37.

### (3) Experts’ evaluation of digital watermarking privacy protection methods and traditional passive privacy protection methods

**Fig 6:**
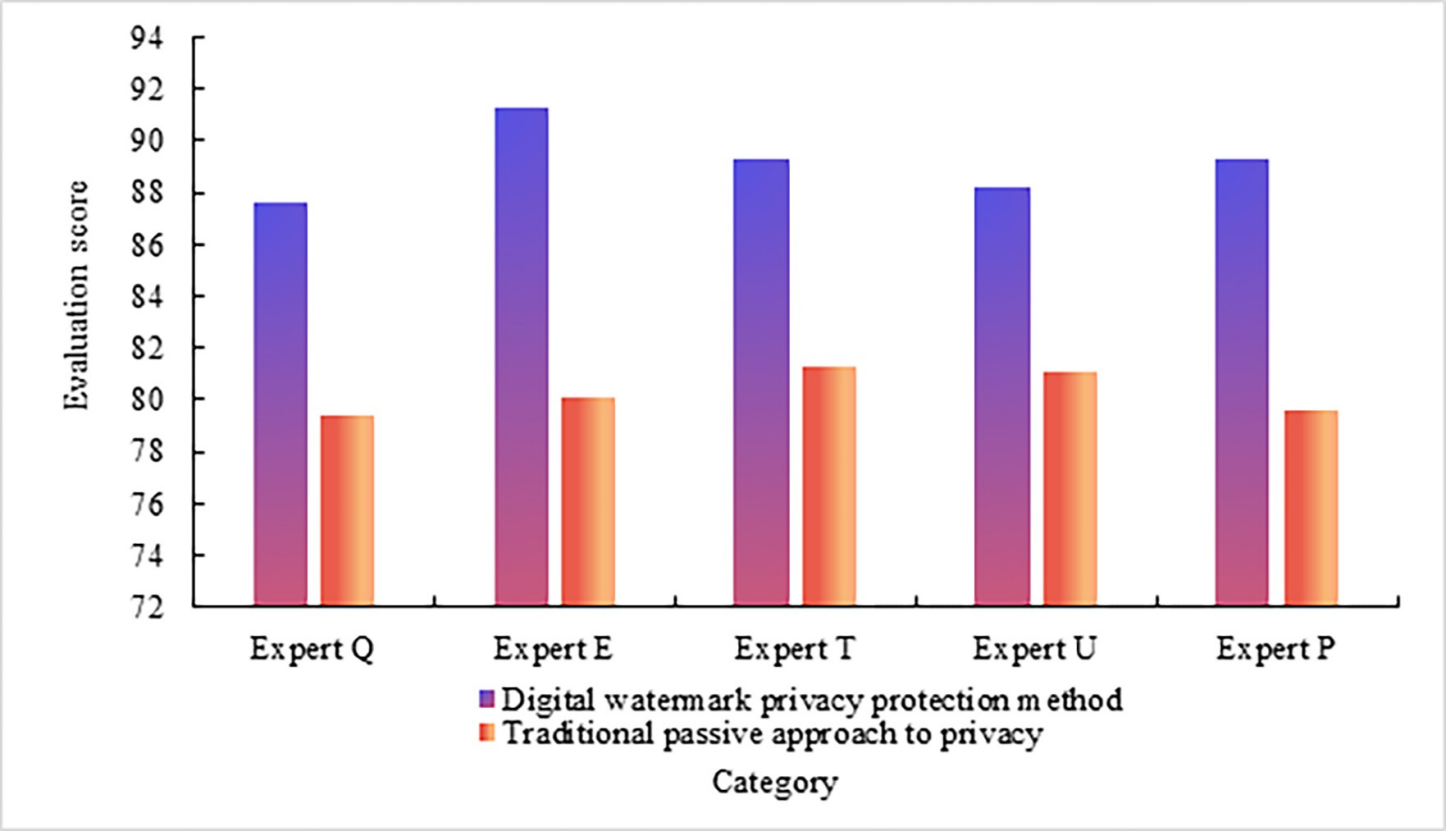


The five experts are called expert Q, expert E, expert T, expert U, and expert P. The evaluation of experts on digital watermark privacy protection methods and traditional passive privacy protection methods is obtained. The evaluation score range is 1–100, as shown in Fig 6 Expert evaluation of digital watermark privacy protection method and traditional passive privacy protection method.

As shown in Fig 6, on the whole, experts’ evaluation scores of digital watermark privacy protection methods are higher than those of traditional passive privacy protection methods. From the analysis of specific data, expert Q’s evaluation score of digital watermark privacy protection method is 87.59, and expert Q’s evaluation score of traditional passive privacy protection method is 79.35. The evaluation score of expert Q on the digital watermark privacy protection method is 8.24 higher than that of expert Q on the traditional passive privacy protection method. Expert E’s evaluation score of digital watermark privacy protection method is 91.26, and expert E’s evaluation score of traditional passive privacy protection method is 80.04. Expert E’s evaluation score of digital watermark privacy protection method is higher than expert E’s evaluation score of traditional passive privacy protection method. Expert T’s evaluation score of digital watermark privacy protection method is 89.32, and expert T’s evaluation score of traditional passive privacy protection method is 81.24. The gap between the two is obvious. Expert U’s evaluation score of digital watermark privacy protection method is 88.16, and expert U’s evaluation score of traditional passive privacy protection method is 81.03. The evaluation score of expert U on the digital watermark privacy protection method is 7.13 higher than that of expert U on the traditional passive privacy protection method. The evaluation score of expert P on the digital watermark privacy protection method is 89.27, and the evaluation score of expert P on the traditional passive privacy protection method is 79.58. The difference between the two is 9.69. From the average data, the average evaluation score of the five experts on the digital watermark privacy protection method is 89.12, and the average evaluation score of the five experts on the traditional passive privacy protection method is 80.25. The average evaluation score of the five experts on the digital watermark privacy protection method is 8.87 higher than the average evaluation score of the five experts on the traditional passive privacy protection method, and the higher proportion is 11.05%. The above data shows that compared with traditional passive privacy protection methods, digital watermarking privacy protection methods have more advantages in protecting the privacy of public service advertising visual design.

### (4) Evaluation on privacy protection methods of public service advertising visual design works push

**Fig 7:**
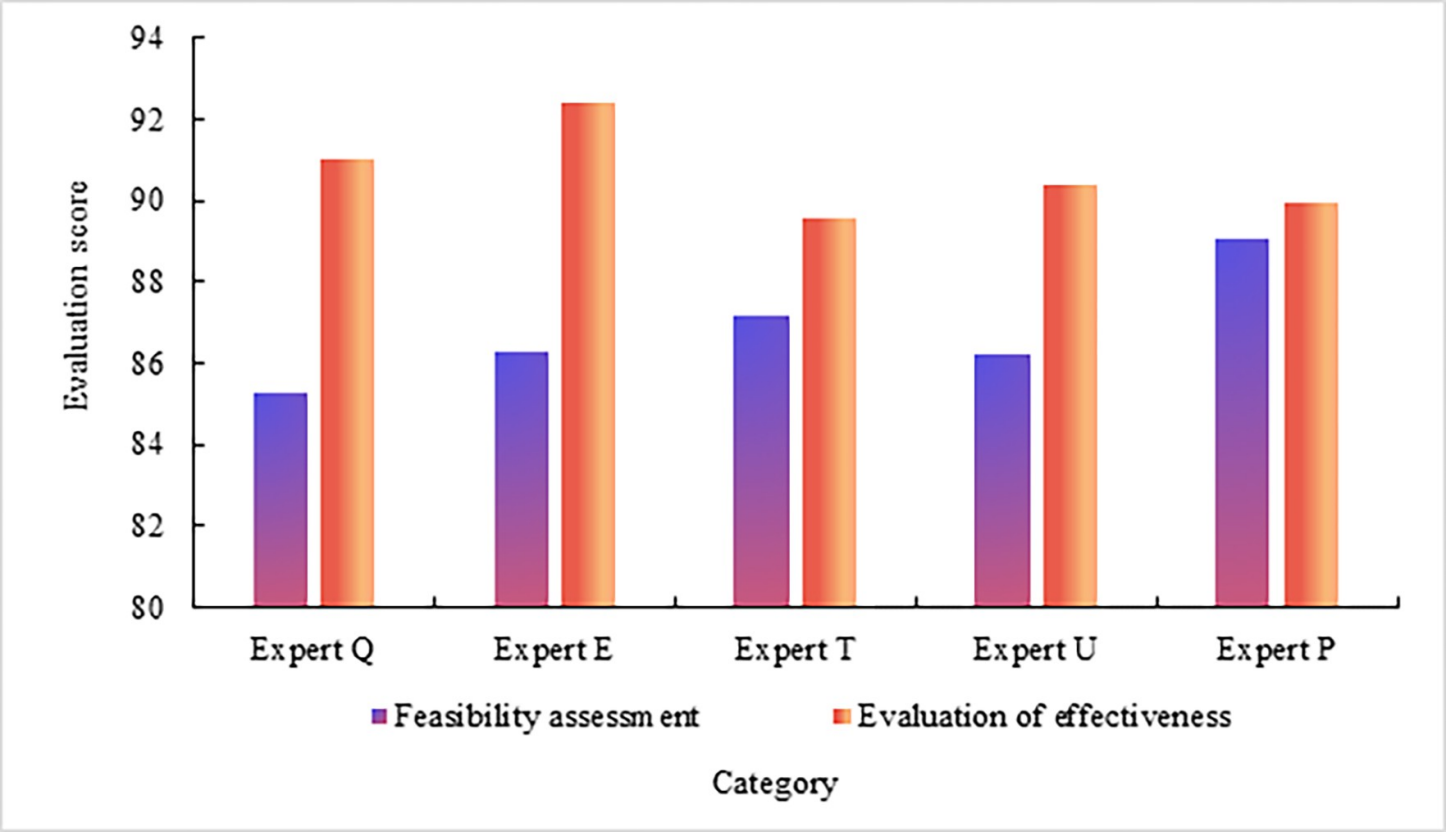


Experts Q, E, T, U and P are invited to evaluate the privacy protection methods pushed by public service advertising visual design works. The evaluation score range is 1–100, as shown in Fig 7 Evaluation of privacy protection methods for the push of visual design works of public service advertisements.

As shown in Fig 7, expert Q’s evaluation score on the feasibility of privacy protection methods pushed by public service advertising visual design works is 85.26, and the evaluation score of the effectiveness of the privacy protection method for pushing public service advertising visual design works is 91.02. Expert E’s evaluation score on the feasibility of the privacy protection method pushed by public service advertising visual design works is 86.31, and the evaluation score of the effectiveness of the privacy protection method for pushing public service advertising visual design works is 92.37. Expert T’s evaluation score on the feasibility of privacy protection methods pushed by public service advertising visual design works is 87.15, and the evaluation score of the effectiveness of the privacy protection method for pushing public service advertising visual design works is 89.57. Expert U’s evaluation score on the feasibility of the privacy protection method pushed by public service advertising visual design works is 86.23, and the evaluation score of the effectiveness of the privacy protection method for pushing public service advertising visual design works is 90.36. Expert P’s evaluation score on the feasibility of privacy protection method pushed by public service advertising visual design works is 89.03. The evaluation score of the effectiveness of the privacy protection method for pushing public service advertising visual design works is 89.96. The average feasibility evaluation score of the experts on the privacy protection method of pushing public service advertising visual design works is 86.8, and the average effectiveness evaluation score of the experts on the privacy protection method of pushing public service advertising visual design works is 90.66. From the above data, it can be seen that the privacy protection method for the push of public service advertising visual design works has certain feasibility and effectiveness.

## 7.Discussion

From the perspective of artificial intelligence Internet of Things, this study successfully put forward the privacy protection methods of digital watermarking and public service advertising works, which provided innovative solutions to solve the privacy problems in public service advertising visual design. The privacy protection method of public service advertising works has also been recognized by experts, and its feasibility and effectiveness evaluation are 86.8 and 90.66 respectively. This means that this method has good feasibility and effectiveness in the actual push process, which can effectively protect the private information involved in advertisement push and make public service advertisements more secure and credible. This study has made remarkable achievements in the field of public service advertising, provided feasible privacy protection solutions, and comprehensively discussed the advantages, limitations and future development direction of the method in the discussion, which made valuable contributions to the research and practice in this field. This paper solves the privacy problem of public service advertisements, and achieves remarkable results through digital watermarking and push privacy protection methods, which improves privacy security and provides innovative solutions for advertising design.

## 8.Conclusions

This paper analyzed the visual elements of public service advertising visual design, and summarized the principles of public service advertising visual design. The privacy protection method for the visual design of public service ads was proposed. After the experimental study on the privacy protection of the visual design of public service ads, the following conclusions were drawn: The awareness of privacy protection of public service advertising visual designers and public service advertising audience needed to be improved; compared with the traditional passive privacy protection method, the digital watermark privacy protection method was more effective in protecting the privacy of public service advertising visual design; the privacy protection method of pushing public service advertising visual design works had certain effectiveness. The shortcoming of this paper is that the potential influence of digital watermarking on image quality is not discussed in depth. In the future, we can expand the research, deeply study the practical application of privacy protection technology and optimize the digital watermarking algorithm to adapt to different scenarios and platforms. Further study the applicability of digital watermarking in different advertising media, deeply analyze the balance between privacy protection and visual effects, and improve the robustness of digital watermarking by combining deep learning and other technologies. Expand to other art forms, such as video advertising, in order to fully understand the practical application of digital watermarking in the advertising field.
